# Affective Entry Characteristics Towards Mathematics and Reflective Thinking Skills Towards Problem Solving: The Mediating Role of Critical Thinking

**DOI:** 10.3390/jintelligence14070149

**Published:** 2026-07-17

**Authors:** Nejla Gürefe, Nursena Evrensel Yumrutepe

**Affiliations:** 1 Mathematics Education, Mersin University, Mersin 33100, Turkey; 2 Mathematics Education, Ministry of Education, Istanbul 34560, Turkey; nur.sena42gs@hotmail.com

**Keywords:** critical thinking tendency, affective entry characteristics toward mathematics, reflective thinking skills for problem solving

## Abstract

The examination of thinking skills has become a contemporary and imperative focus within educational research, capturing considerable attention from scholars. The present study endeavors to explore the mediating role of critical thinking tendency in the correlation between affective entry characteristics toward mathematics and reflective thinking skills related to problem-solving. This study was designed as quantitative research under the cross-sectional survey research design. The sample comprises 649 middle school students, with data collected through the Affective Entry Characteristics Towards Mathematics Scale, Critical Thinking Tendency Scale, and Reflective Thinking Skills Towards Problem Solving Scale, administered via an online survey platform. Mediation analyses were conducted. Results suggested statistically significant indirect associations among these constructs, with critical thinking tendency identified as a partial mediator between affective entry characteristics toward mathematics and reflective thinking skills for problem-solving. These findings suggest a complex interplay among these constructs, warranting further investigation in future studies.

## 1. Introduction

The rapid advancement of science and technology has elevated expectations for individuals and institutions to unprecedented levels. The demand is no longer for individuals who merely memorize information but for those who can interpret it, generate new ideas, exhibit curiosity, pose questions, and engage in purposeful thinking. Particularly, 21st-century skills strive to equip students with competencies such as problem-solving and critical thinking. In the context of educational research, these competencies find their most critical application within mathematics education, where abstract conceptualization and non-routine problem-solving require advanced cognitive control (National Council of Teachers of Mathematics [NCTM], 2020). Within mathematics education, critical thinking, among the most crucial of these skills, empowers individuals to discern right from wrong by making sense of presented information (Ennis, 1985). When applied to mathematical tasks, critical thinking manifests as mathematical reasoning, conjecturing, and validating mathematical arguments (Su et al., 2016). This process represents a sophisticated form of thinking that allows for the interpretation and structuring of knowledge stored in the mind (Lewis & Smith, 1993). Moreover, critical thinking aids individuals in steering clear of attitudes that may negatively impact the thinking process, providing control over their thought processes (Paul & Elder, 2013). Ennis (1985) defined critical thinking as a form of reflective thinking that influences decision-making related to an individual’s beliefs and behaviors. Reflective thinking provides an environment in which students can make thoughtful and logical decisions when faced with complex challenges (Procter, 2020) and contributes to improving students’ accuracy and concentration when dealing with mathematical tasks (Farnila et al., 2021). Questioning mathematics learning process: Since it includes the elements of inter-conceptual association, strategy selection, and reflection (Kramaski et al., 2013), it has been shown that reflective thinking supports meaningful learning in mathematics (Inoue & Buczynski, 2011). Reflective thinking plays an important role in evaluating the internal questioning towards a problem and the efficiency of various mathematical strategies in the problem-solving process (Kramaski et al., 2013). For middle school students transitioning from concrete to formal mathematical operations, reflective thinking skills for problem-solving can be considered vital, as they act as a catalyst enabling students to examine, evaluate, and motivate themselves in the problem-solving process (Kholid et al., 2020; Ramdani et al., 2019). Research suggests the potential for developing critical thinking skills in mathematics (Firdaus et al., 2015; León, 2015) and reflective thinking skills in mathematics (Kember, 2010; Pavitola & Rieksta, 2025). Consequently, it becomes crucial to identify and statistically model the factors influencing the cultivation of these skills.

Given that reflective thinking is predominantly observed in the problem-solving process (Kuncoro & Hidayat, 2025; Shermis, 1992) and that both critical and reflective thinking are essential for academic success in problem-solving situations (Scott & Markert, 1994), it is deemed imperative to ascertain the predictive variables impacting the acquisition of these skills. However, a significant gap remains in the mathematics education literature regarding what these specific predictive variables are and how they interact structurally (Kuncoro & Hidayat, 2025; Manalo et al., 2013; Ormrod, 2018). While general education literature suggests a theoretical link between critical thinking and reflective thinking, there is a lack of empirical, structural models explaining the extent to which reflective thinking skills for problem solving (RTSPS) can be explained by critical thinking tendency within the domain of middle school mathematics.

Furthermore, quantitative mathematics education literature highlights that cognitive outcomes and higher-order thinking skills are significantly predicted by affective factors. In mathematics, these affective entry characteristics are primarily operationalized as interest, attitude, and academic self-concept towards mathematics (Anderson & Bourke, 2000). Many middle school students develop mathematical anxiety or low self-concept, which acts as a psychological barrier, directly hindering their critical (Arpin et al., 2015) and reflective engagement with mathematical problems (Maloney & Retanal, 2020). Despite the consensus that affective characteristics influence mathematics achievement (Pantziara & Philippou, 2015) current research often treats affective variables and higher-order cognitive skills (like critical and reflective thinking) as isolated constructs. It can be argued that there is a significant lack of empirical research that critically examines how students’ affective entry characteristics towards mathematics simultaneously shape their critical thinking tendencies and reflective thinking skills during mathematical problem-solving.

To address these gaps in the literature through a structural approach, this study investigates the extent to which affective entry characteristics—operationalized as interest, attitude, and academic self-concept—predict these skills. Crucially, this study tests a novel structural mediating model where critical thinking tendency acts as a cognitive bridge in the relationship between affective entry characteristics towards mathematics and reflective thinking skills for problem solving. Quantifying these structural relationships among middle school students is methodologically essential for providing generalizable data and explanatory models to those who aim to raise better thinking individuals in the field of mathematics education. In this sense, the study sought answers to the following strictly quantitative research questions:

- Do affective entry characteristics towards mathematics positively and significantly predict reflective thinking skills for problem solving in mathematics?
- Do affective entry characteristics towards mathematics predict critical thinking disposition positively and significantly?
- Do critical thinking tendencies positively and significantly predict reflective thinking skills for problem solving in mathematical contexts?
- Do critical thinking tendencies have a significant mediating role in the relationship between affective entry characteristics toward mathematics and reflective thinking skills for problem solving among middle school students?
- Do the sub-dimensions of the scales meaningfully predict each other?

### 1.1. Critical Thinking

Critical thinking, classified among higher-order cognitive skills, poses a challenge in establishing a standardized definition due to its inherent psychological complexity (Liu & Páztor, 2022). At its most general level, classical frameworks conceptualize critical thinking as the active scrutinization of the logicality, validity, or accuracy of statements (Paul, 1990) which manifests as a reasoned, reflective, and adept cognitive process focused on decision-making regarding beliefs or actions (Nosich, 2012). Considering its definition, critical thinking can be said to be important in mathematics education because it enables students to improve their ability to find solutions to real-life problems and make decisions using the knowledge they have gained in class.

The contemporary empirical research warns against treating critical thinking as a monolithic cognitive trait, emphasizing that its operationalization requires a strict conceptual distinction among its cognitive, affective, and metacognitive dimensions (Halpern & Dunn, 2021). The cognitive dimension, which entails delineating situations, analyzing diverse perspectives, and drawing logical inferences based on evidential support (Bruning et al., 1995; Fisher & Scriven, 1997), must be explicitly distinguished from the affective dimension, commonly operationalized as critical thinking tendency (Facione & Facione, 1992). While the cognitive aspect represents the capacity for evaluating reasoning the affective dimension reflects the psychological effort, intellectual curiosity, and tolerance for ambiguity required to execute that capacity (Suárez et al., 2018). There is also an active metacognitive component that enables creating reflective and action-oriented judgments by combining the overlapping skills, tendencies and knowledge of these two dimensions (Vendrell & Rodríguez, 2020). From a structural standpoint, metacognition acts as the executive governor of thought, enabling individuals to self-reflect, monitor cognitive errors, and strategically deploy mental repertoires during complex problem-solving (Sternberg, 1999). It has been said that critical thinking is characterized by a strong intrinsic drive to use critical thinking to solve problems and make judgments (Madadkhani & Nikoogoftar, 2015). In the current study, the affective dimension of critical thinking is discussed, and research emphasizes that high cognitive capacity can only be transformed into critical thinking when supported by emotional tendencies (Najafi et al., 2022; Wolters, 2003; Zhang, 2003).

Although the instrumental role of critical thinking in ensuring academic success (Sayaf et al., 2022; Kazemi et al., 2020) and driving conceptual change has long been established, traditional literature has frequently examined this construct in isolation. A prominent gap remains in the literature regarding how these explicit dimensions of critical thinking simultaneously interact with domain-specific affective barriers (such as mathematics anxiety) to shape reflective thinking during complex task execution. Most existing studies either examine affective variables or high-order cognitive skills as isolated constructs, leaving a critical empirical void concerning their structural interplay. To address this limitation, the present study specifically delves into the examination of the mediating role played by students’ critical thinking tendencies in relation to these interconnected affective and cognitive variables.

### 1.2. Affective Entry Characteristics Toward Mathematics

A student’s emotional inclinations toward a course constitute their affective characteristics (Bloom, 1998). While traditional frameworks view emotion and cognition as isolated domains, contemporary research considers mood as a dynamic catalyst that directly influences learning and achievement (Edirmanasinghe, 2020; Ganley & Lubienski, 2016; Samuelsson, 2023). Within this interplay, affective traits dictate the mental resilience and working memory capacity students deploy when facing rigorous mathematical challenges (Bloom, 1998).

Bloom (1998) theoretically conceptualizes affective entry characteristics as a triad of interest, attitude, and academic self-concept. To improve the conceptual distinctions within empirical models, these key constructs must be clearly delineated: *interest* represents an intrinsic, situation-specific curiosity toward activities (Sevgi & Alpaslan, 2020); *attitude* is a stable, longitudinal evaluative tendency toward the subject (Papanastasiou, 2002); whereas *academic self-concept* operates at a meta-cognitive level as the global belief in one’s prowess. Although distinct in early childhood, these variables progressively overlap during adolescence, coalescing into a single, integrated latent factor that justifies its unified operationalization in predictive modeling (Çalışkan & Serçe, 2016).

Crucially, a robust affective foundation frees students from cognitive anxiety, enabling higher-order behaviors such as reflective thinking for problem-solving (monitoring steps and analyzing errors) and critical thinking (questioning assumptions and self-regulation) (Arpin et al., 2015; Maloney & Retanal, 2020). Student affect may not automatically guarantee reflective problem-solving unless it first triggers the analytical and evaluative behaviors inherent in critical thinking, positioning the latter as a vital structural mediator.

The existing paradigm remains largely descriptive and direct, repeatedly confirming that affect matters while leaving the internal explanatory mechanisms unexamined. Specifically, how mathematics affect concurrently drives reflective problem-solving and critical thinking tendency within a unified framework remains unexplored. Furthermore, the explicit mediating role of critical thinking tendency between affect and reflective problem-solving is empirically untested. This study addresses this gap by testing a structural mediation model to map how affective entry characteristics influence reflective thinking for problem-solving through the mediating mechanism of critical thinking tendency.

### 1.3. Reflective Thinking Skills for Problem Solving

Reflective thinking toward problem-solving is a metacognitive monitoring mechanism in which an individual filters past experiences through an analytical lens, evaluates their current cognitive schemas, and transforms and uses structured information to overcome complex challenges (Farnila et al., 2021; Kholid et al., 2020; Rodgers, 2002). Classical educational psychology frameworks have traditionally positioned reflective thinking as either a direct substitute for or a linear precursor to critical thinking (King & Kitchener, 1994; Phan, 2009). However, current empirical trends clarify the conceptual boundaries between these two constructs, conceptualizing reflective thinking not as an isolated cognitive skill, but as a higher-level umbrella mechanism that regulates how multidimensional structures (cognitive, metacognitive, and affective) emerge in problem-solving processes (Merkebu et al., 2023). n this conceptual distinction, the interaction of critical and reflective thinking skills points to a dynamic functionality. Reflective thinking is a pathway of “internal questioning and awareness” that enables the learner to actively discriminate existing information and identify cognitive gaps and obstacles (Afshar & Rahimi, 2016). When the student gains control over their own cognitive processes through this internal questioning, they reach the potential to self-correct and evaluate alternative solutions to overcome the mathematical obstacles they encounter (Dubinsky, 2002). At this point, it can be said that critical thinking tendency functions as an empirical catalyst integrated into the reflective thinking process. Critical thinking deepens cognitive processing by filtering data logically, evaluating arguments, and making strategic decisions, enabling the individual to make the most appropriate move towards the goal, to pause and reason (Nosich, 2012). Therefore, students who become proficient in reflective thinking are better equipped to activate and structure critical thinking mechanisms in problem-solving contexts (Kuncoro & Hidayat, 2025).

Although reflective thinking is known to support innovative problem-solving by triggering higher-level processes such as abstraction, logical proof generation, and iterative solution refinement (Tall, 2002), the current literature has not sufficiently explored this structural synergy. While the relationship between reflective thinking and academic achievement and cognitive processes has been widely examined in general education frameworks (Chamdani et al., 2022), these studies have mostly been limited to descriptive descriptions. Given that the affective dimension, an integral part of students’ cognitive processes, is also involved, there is a need for empirical research that tests this structure with a holistic and structural model. Current empirical studies show that students with strong reflective thinking skills possess greater emotional resilience and intellectual curiosity in counteracting mathematical anxiety and managing conceptual ambiguities (Maloney & Retanal, 2020). These emotionally receptive and positive affective input characteristics allow students to systematically analyze complex mathematical structures.

However, in the current mathematics education literature, these structures—affective entry characteristics, critical thinking tendency, and reflective thinking—have been largely studied as isolated, independent variables. There is a noticeable lack of empirical research in the literature that tests, using a holistic and structural model, how students’ affective entry characteristics, such as intrinsic motivation and math anxiety, shape their higher-level reflective thinking processes towards problem-solving, and how critical thinking tendency acts as a mediating channel in this complex relationship. The literature’s inability to capture this nonlinear, multidimensional interaction leaves the mechanism of the transformation of latent emotional tendencies into active mathematical reflection obscure. This study aims to address this critical limitation by testing the structural and mediating role of critical thinking tendencies in the relationship between students’ affective profiles and their reflective thinking capacities, and to present a holistic, nonlinear, and original theoretical model for mathematical meaning-making and problem-solving processes.

## 2. Method

### 2.1. Model of the Research

This study was designed as quantitative research under the cross-sectional survey research design, which is highly appropriate for examining trends and relationships among a specific population at a single point in time (Creswell, 2012). Within this framework, a correlational and mediation analysis model was utilized as the primary data analysis technique to investigate whether there exists a significant mediating variable—critical thinking tendency—in the correlation between affective entry characteristics toward mathematics and reflective thinking skills for problem solving among middle school students. To ensure ethical standards, official permissions for the utilization of the scales were secured from the authors, and formal ethics committee approval was obtained prior to the data collection process. A convenience sampling method was employed for participant recruitment due to its accessibility and logistical feasibility, and participation was entirely voluntary. To strictly adhere to ethical guidelines regarding underage participants, a clear distinction was maintained between parental consent and student assent. Before the administration, written parental consent was obtained from the students’ legal guardians. Additionally, student assent was secured by informing the students about the study’s purpose and obtaining their voluntary agreement to participate. The survey instrument was then administered electronically via an online data collection platform.

The data collection process was implemented in two distinct settings to accommodate student accessibility: First, a controlled administration was conducted during regular school hours, where students completed the online forms in the school computer laboratories under the supervision of the researchers and classroom teachers. Second, for students who were unable to complete the forms during school hours, the online survey link was shared directly with their parents. These parents were thoroughly informed about the study’s purpose through an information sheet and requested to supervise their children while completing the forms at home to ensure data integrity and prevent external distractions.

### 2.2. Participants

The target audience of this research is students studying in middle school. The sample includes 649 middle school students from a province in the Marmara Region of Turkey. Regarding the institutional characteristics of the research environment, the data were collected from public, co-educational (mixed-gender) middle schools located in urban areas. The student population in these schools predominantly represents a middle to lower-middle socioeconomic status, reflecting the typical demographic profile of public school students in the region. In this study, a non-probability convenience sampling method was employed to select the participants. The study was conducted with middle school students who were accessible to the researchers and who participated on a strictly voluntary basis. Although data were collected from predefined classes within the accessible schools, the final sample was determined by student availability, parental consent, and student assent, rather than a probabilistic random or systematic selection. Therefore, the sampling approach is defined strictly as convenience sampling to ensure methodological accuracy. This approach allows researchers to select participants who are close and easy to reach to enhance speed and practicality during the data collection process (Merriam, 2009). The demographic information of the 649 students in the study is presented in Table 1. The overall mean age of the participants was calculated as 12.14, when analyzed by gender, the mean age was 12.11 for female students and 12.17 for male students. Furthermore, regarding the grade levels, the mean age was determined as 10.65 for 5th graders, 11.62 for 6th graders, 12.68 for 7th graders, and 13.63 for 8th graders.

In addition, since the study employed a cross-sectional research design, the findings reflect relationships observed at a single point in time and therefore do not allow for causal inferences between variables.

### 2.3. Data Collection Tools and Process

As data collection tools in the study, three different scales (look at Appendix A) were used. These scales are “Affective Entry Characteristics for Mathematics”, “Critical Thinking Tendency” and “Reflective Thinking Skills for Problem-Solving”. Furthermore, we employed a personal information form encompassing class and gender details of the students. The scales utilized in the study were specifically developed for middle school students. All three of these scales were in Turkish form and the original versions of these scales were utilized without any alterations. Data were gathered from 649 middle school students, and their validity and reliability were subjected to analysis. In this study, the reliability of the scales was calculated using Cronbach’s Alpha coefficients. For construct validity, Confirmatory Factor Analysis (CFA) was performed to test whether the factorial structures of the scales exhibited acceptable fit for our study sample, and fit indices were examined.

*Affective Entry Characteristics Toward Mathematics* scale was developed by Çalışkan and Serçe (2016) specifically for the Turkish educational context to measure students’ non-cognitive internal factors regarding mathematics. The primary purpose of this scale is to evaluate middle school students’ multidimensional affective states, including their interest, attitudes, and academic self-concept toward mathematics. The scale consists of 20 positively framed items, utilizing a 4-point Likert-type rating scale that ranges from “Strongly Agree” (1-point) to “Strongly Disagree” (4-point). The total score obtainable from this scale ranges from a minimum of 20 to a maximum of 80, where a higher score signifies a more positive affective orientation toward mathematics. Sample items from the scale include statements such as “Studying mathematics comes easily to me.”, “I am confident that I can learn the topics in math class” and “I am interested in mathematics.”. The factor structure of the scale was established through confirmatory factor analysis, where the confirmatory factor analysis results were as follows: X^2^/df = 2.525; *p* = .000 < .001; RMSEA = 0.059; CFI = 0.768; GFI = 0.903; AGFI = 0.876. It was confirmed that the scale’s factors explained 50.129% of the variance. Moreover, the Cronbach’s Alpha value for the scale was calculated as 0.947. In the context of our research, we performed a confirmatory factor analysis on the scale, and the obtained results were congruent with the original form of the scale (X^2^/df = 2.9; *p* = .000 < .001; RMSEA = 0.068; CFI = 0.96; GFI = 0.92; AGFI = 0.88). The Cronbach’s Alpha value for our study was determined as 0.963, indicating high internal consistency. The scale comprises one factor.

*Critical Thinking Tendency* scale was developed by Yıldırım Döner and Demir (2022) within the Turkish cultural context to assess the psychological and cognitive orientations of the participants toward logical reasoning. The main purpose of this scale is to determine middle school students’ inclinations, openness, and habits of mind regarding critical thinking in various contexts. It comprises 21 positively framed items, utilizing a 5-point Likert-type rating scale that ranges from “Never” (1-point) to “Always” (5-point). Consequently, the overall score for this scale ranges from a minimum of 21 to a maximum of 105, with higher scores reflecting a stronger overall critical thinking tendency. For its specific components, the minimum and maximum possible scores are proportional to the number of items within each sub-dimension. Sample items from the scale include statements such as “I remain impartial when evaluating opposing viewpoints.”, “I can focus on the details of events.” and “I believe critical thinking has benefited me.”. The scale is organized into three sub-dimensions: dialectical thinking, tension, and analysis. The factor structure of the scale was established through confirmatory factor analysis, with the following results: X^2^ = 345.18, sd = 184, RMSEA = 0.042, NFI = 0.97, NNFI = 0.98, CFI = 0.99, GFI = 0.88, AGFI = 0.85, SRMR = 0.059. The variance explained by the factors of the scale was determined to be 42.94%, and the reliability analyses yielded a Cronbach’s Alpha value of 0.87. In the context of our research, a confirmatory factor analysis was conducted, and the obtained results (X^2^/df = 2.79; *p* = .000 < 0.001; RMSEA = 0.053; CFI = 0.93; GFI = 0.93; AGFI = 0.91) indicated that the scale remains consistent with its original form. The Cronbach’s Alpha value for our study was calculated as 0.897, signifying a high level of internal consistency. In summary, the Critical Thinking Tendency scale, with its three sub-dimensions (Dialectical Thinking, Disposition, Analysis), has shown robust psychometric properties in our research, maintaining reliability and consistency with the original scale.

*Reflective Thinking Skills For Problem Solving* scale was developed by Kızılkaya and Aşkar (2009) and formally validated for Turkish students to evaluate the cognitive and metacognitive strategies employed by students during challenging tasks. The primary purpose of this scale is to determine middle school students’ reflective thinking levels specifically within problem-solving contexts across three sub-dimensions: questioning, evaluation, and reason-searching. It consists of 14 positively framed items, utilizing a 5-point Likert-type rating scale that ranges from “Never” (1-point) to “Always” (5-point). The total score that can be obtained from the entire scale ranges from a minimum of 14 to a maximum of 70, where higher scores indicate more advanced reflective thinking skills in problem-solving scenarios. Regarding its sub-dimensions (questioning, assessment, and reasoning), the minimum and maximum scores vary according to the respective item density of each sub-scale. Sample items from the scale include statements such as “When I solve a problem, I review and evaluate the steps I took.”, “When solving problems, I perform each step by considering the steps before and after.” and “When I read a problem, I ask myself questions to determine what is given and what is asked.”. The scale is organized into three sub-dimensions: questioning, assessment, and reasoning. The factor structure of the scale was determined through confirmatory factor analysis, resulting in the following indices: RMSEA = 0.071; X^2^/df = 2.69; *p* = .000 < .001; GFI = 0.92; NNFI = 0.93; CFI = 0.95; AGFI = 0.89. It was established that the variance explained by the factors of the scale was 42.94%, and the reliability analyses yielded a Cronbach’s Alpha value of 0.830. In our research, a confirmatory factor analysis was conducted, and the obtained results (X^2^/df = 3,0; *p* = 0.000 < 0.001; RMSEA = 0.056; CFI = 0.96; GFI = 0.96; AGFI = 0.93) indicated that the scale remains consistent with its original form. The Cronbach’s Alpha value for our study was calculated as 0.905, reflecting a high level of internal consistency. To summarize, our results suggest that the Reflective Thinking Skills for Problem Solving scale exhibits reliable psychometric properties consistent with the original instrument.

The data were collected from middle school students through Google Forms in the spring term of 2021–2022. The scales in the study were created on the Google Forms platform and sent to the parents of students via the Internet. The researcher informed the students and their families about the voluntariness and the confidentiality of the participants. In addition, there was no time limit to answer the items in the scale. It was accepted that middle school students answered the statements on the scale accurately and sincerely.

### 2.4. Data Analysis

Prior to conducting the analysis, we performed extreme value analysis, normality checks, and homogeneity analysis to assess whether the dataset met the prerequisites for parametric studies. In this context, we excluded the data of eight students identified as extreme values from the dataset. Subsequently, we scrutinized the kurtosis and skewness coefficients. Following these examinations, it was determined that the data exhibited a normal distribution. The initial data analysis involved the utilization of Pearson Product-Moment Correlation Analysis to explore the relationships between descriptive statistics and variables. Upon identifying a significant relationship between the variables, we established and tested the mediation model. The mediation model analysis involved testing Model 4 and Model 6 through the utilization of the Process v4.2 extension (Hayes, 2017) within IBM SPSS 26.0 to assess whether critical thinking tendency played a mediating role in the relationship between affective entry characteristics toward mathematics and reflective thinking skills for problem solving. Within the model, the 5000 bootstrapping approach recommended by Preacher and Hayes (2004) was conducted, and a 95% confidence interval was applied. The determination of the significance of effects in the model relied on both *p* values and confidence intervals (CI). Specifically, a *p* value below .05 or the absence of 0 within the confidence intervals was considered indicative of statistically significant variable effects (Hayes, 2009). Various authors have suggested that this method yields more reliable results compared to the traditional Baron and Kenny’s (1986) causal steps method and the Sobel test (Preacher et al., 2007; Zhao et al., 2010).

## 3. Findings

### 3.1. Preliminary Analyses

Before starting the mediation model analysis, we analyzed the data using descriptive statistics and the relationships between dependent, independent and mediator variables conduct correlation (see Table 2).

The findings suggest a significant correlation between affective entry characteristics toward mathematics and critical thinking tendency (r = 0.498, *p* < .05), affective entry characteristics toward mathematics and reflective thinking skills for problem solving (r = 0.582, *p* < .05), and reflective thinking skills for problem solving and critical thinking tendency (r = 0.726, *p* < .05). Correlation coefficients are categorized according to Evans (1996). According to this, a moderately significant positive correlation was found between affective entry characteristics toward mathematics and critical thinking tendency, as well as between affective entry characteristics toward mathematics and reflective thinking skills for problem solving. A highly significant positive correlation was observed between reflective thinking skills for problem solving and critical thinking tendency.

Regarding the normal distribution of the data, kurtosis and skewness coefficients were calculated. The results showed that the skewness of the variables varied between −0.208 and −0.614, and their kurtosis varied between 0.850 and −1.963. George and Mallery (2010) suggested that reference values should be between +2.0 and −2.0 for both kurtosis and skewness. The examination of kurtosis and skewness values in the research structures indicated that they were within the reference values, suggesting a normal distribution of the dataset. In addition to assessing normal distribution, examinations were conducted for multicollinearity and residuals. The Durbin Watson value was determined to be 1.97, while the variance inflation factor values were 1.33, and the tolerance value was 0.75. Upon careful consideration of these results, it was concluded, in accordance with Field (2005), that neither multicollinearity nor residual problems were evident.

### 3.2. The Results of Mediator Analysis

**Figure 1:**
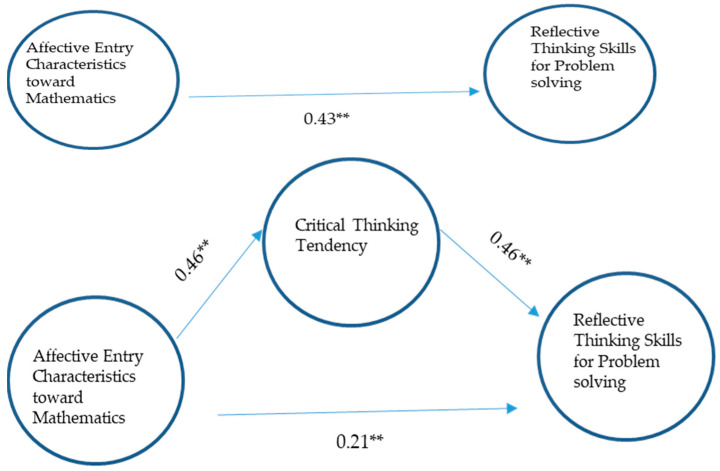
The mediation model (** *p* < .01).

Applying process model 4, we tested whether critical thinking tendency mediated the relationship between affective entry characteristics toward mathematics and reflective thinking skills for problem solving in middle school students (see Figure 1).

According to the analysis, affective entry characteristics toward mathematics positively and significantly predicts both reflective thinking skills for problem solving (β = 0.43, t = 18.18) and critical thinking tendency (β = 0.46, t = 14.61). Additionally, critical thinking tendency also positively and significantly predicts reflective thinking skills for problem solving (β = 0.46, t = 20.03). Interpreting the ß values, it can be observed that in the absence of a mediator variable, reflective thinking skills for problem solving increases by 0.43 standard deviations when affective entry characteristics toward mathematics increases by one standard deviation. This effect was deemed statistically significant (*p* < .01). Additionally, it was identified that reflective thinking skills for problem solving increases by 0.46 when critical thinking tendency increases by one standard deviation. After adding the mediator variable (critical thinking tendency) to the model, the regression coefficient between affective entry characteristics toward mathematics and reflective thinking skills for problem solving was calculated as 0.21, which is lower than in the base model. This reduction was found to be significant (*p* < .01), indicating that critical thinking tendency is partial mediator the relationship between affective entry characteristics toward mathematics and reflective thinking skills for problem solving. To establish the mediating role of critical thinking tendency between affective entry characteristics toward mathematics and reflective thinking skills for problem solving, the bootstrapping procedure suggested by Preacher and Hayes (2004) was employed. Confidence intervals for the indirect effect were calculated based on a sample size of 5000 individuals (see Table 3).

Upon examining the analysis, it is observed that the indirect effect indicating the mediating role of critical thinking tendency in the relationship between affective entry characteristics toward mathematics and reflective thinking skills for problem solving is 0.2166 [CI: LO = 0.18 HI = 0.25]. Statistically, when analyzing the indirect effect within the 95% bootstrap confidence interval, it was found to be different from zero and above zero (between BootLLCI = 0.1815 and BootULCI = 0.2525), indicating statistical significance. In analyses utilizing the bootstrap technique, mediating effect hypotheses are supported only if the lower and upper bound confidence interval values in the 95% confidence interval do not include zero (0) (MacKinnon et al., 2004). This outcome affirms that critical thinking tendency is a mediating variable in the relationship between affective entry characteristics toward mathematics and reflective thinking skills for problem solving. The significance holds true in both the research and bootstrapping samples (5000). In mediation models, another pivotal parameter is the fully standardized size of the indirect effect, denoted as K^2^ = 0.28. In interpreting effect sizes, evaluations are categorized as low effect if K^2^ = 0.01, medium effect if K^2^ = 0.09, and high effect if K^2^ = 0.25 (Preacher & Kelley, 2011). This effect size of K^2^ = 0.28 is considered high according to the criteria set by Preacher and Kelley (2011). Therefore, the analysis leads to the conclusion that critical thinking tendency serves as a substantial and statistically significant mediator in the correlation between affective entry characteristics toward mathematics and reflective thinking skills for problem-solving.

### 3.3. The Results of Mediator Analysis for Sub-Dimensions of Scales

**Figure 2:**
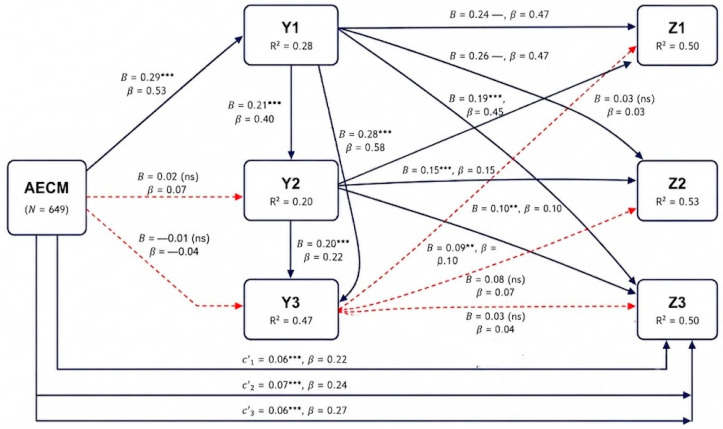
The mediation model for sub-dimensions of scales (** *p* < .01, *** *p* < .001, (ns) *p* > .05).

In the research model, the relationship between the independent variable, affective entry characteristics toward mathematics, and the dependent variable, reflective thinking skills in problem solving, with its sub-dimensions Inquiry (Z1), Reasoning (Z2), and Evaluation (Z3), was tested using the serial multiple mediation role of the mediating variable, critical thinking tendency, with its sub-dimensions Dialectical Thinking (Y1), Disposition (Y2), and Analysis (Y3), using the PROCESS macro (Model 6) developed by Hayes (2022). The analyses were performed with a 95% confidence interval (CI) and a 5000 bootstrap sample size. The sample size was determined as *N* = 649. The visual representation of the analysis is presented in Figure 2.

Due to the hierarchical structure, the predictive levels of the mediating variables among themselves and by the independent variable are common to all models. According to the analysis results, the AECM variable predicts the Y1 mediating variable positively and statistically significantly (B = 0.2945, SE = 0.0185, t = 15.88, *p* < .001, β = 0.5297). This first-stage model explains 28% of the total variance (R^2^ = 0.2805, F(1, 647) = 252.29, *p* < .001). While the direct effect of AECM on Y2 was not found to be significant (B = 0.0211, *p* = .0785), the first mediating variable, Y1, significantly and positively predicted Y2 (B = 0.2097, SE = 0.0215, t = 9.74, *p* < .001, β = 0.4040). Finally, the direct effect of AECM on the third mediating variable, Y3, was insignificant (B = −0.0111, *p* = .2208); however, Y1 (B = 0.2792, *p* < .001, β = 0.5819) and Y2 (B = 0.2014, *p* < .001, β = 0.2178) strongly predicted the Y3 variable. This stage explains 46.9% of the total variance (R^2^ = 0.4691, F(3, 645) = 190.01, *p* < .001).

In the analyses performed for each dependent variable, all total, direct, and specific indirect effects were examined in detail (Table 4).

As shown in Table 4, the total effect of AECM on Z1 (β = 0.5184, *p* < .001) and its direct effect (β = 0.2180, *p* < .001) are significant. Similarly, in the Z2 model, the total effect (B = 0.1566, *p* < .001) and the direct effect (B = 0.0691, *p* < .001) remained significant; and in the Z3 model, the total effect (B = 0.1290, *p* < .001) and the direct effect (B = 0.0632, *p* < .001) retained their significance. These findings indicate that mediating variables play a partial mediation role in the effect of the independent variable on the dependent variables.

Bootstrap indirect effect analyses (5000 resampling) revealed that for all three dependent variable models, only two specific indirect paths out of seven were statistically significant. The single mediation path AECM → Y1 → Z1, Z2, Z3 had significant confidence intervals in all models [95% CI for Z1 = (0.0567, 0.0871); 95% CI for Z2 = (0.0573, 0.0880); 95% CI for Z3 = (0.0438, 0.0687)]. Similarly, the sequential multiple mediation path AECM → Y1 → Y2 → Z1, Z2, Z3 was also found to have a significant indirect effect in all three models with zero-free confidence intervals [95% CI for Z1 = (0.0044, 0.0145); 95% CI for Z2 = (0.0020, 0.0112); 95% CI for Z3 = (0.0013, 0.0095)].

When other unsupported indirect pathways were examined, it was found that all of the bootstrap 95% confidence intervals included the value of zero (0) and were therefore statistically insignificant (see Table 4). This empirically confirms that the successive multiple mediation mechanism does not function through the third mediating variable (Y3), and the dependent variable, reflective thinking skills in problem solving.

In mediation models, another pivotal parameter is the fully standardized size of the indirect effect. In interpreting effect sizes, evaluations are categorized as low effect if K^2^ = 0.01, medium effect if K^2^ = 0.09, and high effect if K^2^ = 0.25 (Preacher & Kelley, 2011). The overall indirect effect of affective entry characteristics toward mathematics on the inquiry dimension was found to be statistically significant with a high effect size (K^2^ = 0.30). This finding showed that affective entry characteristics toward mathematics have a very strong and important indirect role in shaping the Inquiry dimension. In this relationship, the effect size was close to high (K^2^ = 0.24) when Dialectical Thinking alone acted as a mediator, while it decreased to a low level (K^2^ = 0.03) when Dialectical Thinking and Disposition were mediated together. It was determined that affective entry characteristics toward mathematics also had a high effect on the Reasoning dimension (K^2^ = 0.30). In this process, when Dialectical Thinking alone acted as a mediator, the effect of affective entry characteristics toward mathematics showed an effect close to high (K^2^ = 0.24). In contrast, when Dialectical Thinking and Disposition acted as mediators, the indirect effect on the Reasoning dimension decreased to a low level (K^2^ = 0.02). It was determined that the affective entry characteristics toward mathematics also had a high effect on the Evaluation dimension (K^2^ = 0.27). In this relationship, while Dialectical Thinking was the mediating variable, affective entry characteristics toward mathematics continued to have a high effect on the Evaluation dimension (K^2^ = 0.23), but when Dialectical Thinking and Disposition acted as mediators, the effect on Evaluation decreased to a low level (K^2^ = 0.02). No significant effect was observed in any of the other ways.

## 4. Conclusions

In accordance with the research findings, it has been concluded that the affective entry characteristics of middle school students towards mathematics significantly and positively predict their reflective thinking skills for problem-solving. This empirical finding is consistent with the model’s first research question, suggesting that the internal emotional and evaluative ecosystem a student brings to the mathematics classroom is not a passive byproduct, but is closely associated with how they monitor and evaluate their own problem-solving steps. By directly anchoring this result to our statistical indicators, the predictive power observed in this study extends the classical assertions of researchers (Bloom, 1998; Maass & Schlöglmann, 2009; Samuelsson, 2023), who theoretically linked affective traits to baseline learning velocity and effort. However, whereas historical literature often leaves this relationship as a direct, unmediated correlation with general achievement (Anderson & Bourke, 2000; Seel, 2012), our data uncovers a more nuanced, process-oriented cognitive implication: positive affect actively unlocks the meta-cognitive resources required for *reflective thinking*—such as error analysis, strategy evaluation, and self-awareness during problem-solving (Ünver, 2003). When students possess a high academic self-concept their working memory is liberated from the cognitive load of mathematics anxiety (Ashcraft & Kirk, 2001). An individual experiencing math anxiety will be more prone to avoidance and will spend less time trying to solve the problem, especially if the individual is metacognitively aware of the negative emotion (Scheibe et al., 2023), while individuals with high self-efficacy perceptions are more determined, more diligent, more persistent, and more positive when dealing with difficult learning tasks (Zimmerman, 2000). Another important finding obtained in line with the research is that the affective entry characteristics of middle school students towards mathematics significantly and positively predict their critical thinking tendencies. This statistical path confirms that the student’s emotional readiness, intrinsic motivation, and academic self-concept are the primary psychological triggers that activate higher-level analytical questioning before or during mathematical tasks. Bloom (1998) defines affective entry characteristics as a composite of interest, attitude, and academic self-concept; he argues that this composite constitutes the source of learning motivation (Özçelik, 1998) and determines the effort to be shown in the learning process. Critical thinking tendency, on the other hand, is the individual’s proactive willingness to use cognitive strategies, make inferences, and produce logical explanations (Halpern, 1998). Traditional descriptive education literature tends to treat the affective domain and critical cognition as parallel but separate structures. However, our predictive data are consistent with a significant association between these two domains. Critical thinking is not a spontaneous, mechanical process; as literature suggests, it tends to require a high level of mental effort and intellectual courage (Ennis, 1991). Individuals with positive affective input characteristics tend to be more attentive, persistent, and successful in the learning process (Anderson & Bourke, 2000). In the context of mathematics education, this emotional confidence directly provides the psychological foundation necessary for students to actively question mathematical propositions, demonstrate resilience in the face of complex situations, and develop alternative solutions by reducing math anxiety (Daud & Santoso, 2018).

Another important finding from our research is that middle school students’ critical thinking tendencies significantly and positively predict their reflective thinking skills toward mathematical problem-solving. This empirical evidence strongly supports the third research question, which suggests that an analytical and evaluative mindset serves as a fundamental cognitive engine for systematic and retrospective cognitive monitoring processes during the execution of mathematical problem-solving tasks. The current literature frequently highlights the mutual enrichment and shared cognitive foundations between these two higher-level processes (Leung & Kember, 2003; Mezirow, 1991; Phan, 2009). The fact that reflective thinking acts as a catalyst enabling students to examine, evaluate, and motivate themselves in the problem-solving process (Kholid et al., 2020; Ramdani et al., 2019), and that critical thinking overlaps with questioning, analysis, and evaluation skills, points to parallel mental operations and parallels Ennis’s (1985) fundamental claim that critical thinking is inherently reflective and logical. Furthermore, the fact that the functional stages of reflective thinking include processes such as defining the problem, evaluating possible solutions, experimenting, and accepting or rejecting the results (Lee, 2005) clearly reveals how intricately this structure is intertwined with the framework of mathematical problem-solving. However, a deeper and more critical synthesis of our statistical data requires us to framework the structural orientation and task-specific nature of this relationship, as the precise interaction pathway of these two concepts has invited various interpretations in the literature. While some alternative empirical perspectives suggest that reflective actions can precede or shape broader critical thinking behaviors (Aryani et al., 2017; Erdogan, 2019; Ghanizadeh, 2017), our empirical findings firmly validate a unidirectional predictive pathway where critical thinking tendency operates as an essential precursor to reflective problem-solving (Göğüş et al., 2020). This directional alignment is strongly justified by the cognitive demands of mathematical problem-solving, which is inherently a complex and multifaceted process requiring various solution approaches (Daud & Santoso, 2018). Students must first activate a critical inclination to use cognitive strategies, make inferences, and evaluate possible scenarios (Halpern, 1998; Sasson et al., 2018). Without establishing this initial critical framework to systematically break down and filter the problem, the retrospective and metacognitive evaluation process—the core characteristic of reflective thinking—cannot be effectively triggered. Therefore, our data conclusively clarifies this conceptual distinction: critical thinking tendency functions as the active, immediate analytical filter during problem-solving, which systematically paves the way for reflective thinking to serve as the retrospective evaluator of those executed analytical steps.

This study concludes that middle school students’ critical thinking tendencies significantly and positively mediate the relationship between their emotional entry characteristics toward mathematics and their reflective thinking skills for problem-solving. The data support a statistically significant indirect association between emotion and metacognitive reflection, suggesting that this link is mediated by an analytical and evaluative cognitive engine rather than functioning as a direct transmission line. While studies examining these three constructs simultaneously within a unified mediation model are lacking, each variable has frequently been investigated in individual research efforts. Critical thinking tendency involves systematic questioning, logical evaluation, and rational explanations (Halpern, 2014; Rodd, 1999); while reflective thinking involves evaluating assumptions and monitoring strategic consequences in the problem-solving process (Ghanizadeh, 2017). However, a critical synthesis of our mediation data requires us to problematize how these concepts interact hierarchically. Traditional descriptive literature often creates conceptual confusion by suggesting that critical thinking tendency is merely a higher, more conscious extension of reflective thinking (Leung & Kember, 2003). Our empirical findings challenge this nested perspective and support a clear directional ordering: Emotional input characteristics (interest, attitude, and academic self-concept) function as the primary psychological energy that reduces anxiety and fuels cognitive engagement (Hong & Choi, 2015). According to our findings, while this emotional energy alone triggers reflective problem-solving, this triggering is even greater when the positive emotional state is primarily functionalized by the analytical and criterion-oriented nature of critical thinking tendencies. Critical thinking tendency acts as a structured pipeline that transforms vague emotional willingness into an organized, self-regulated, and retrospective reflective follow-up. Since emotional traits, critical thinking tendencies, and reflective skills are all captured by self-report scales, our mediation model maps the structural relationships between perceived internal states and perceived cognitive workflows. Therefore, our findings suggest that critical thinking tendency acts as a statistically significant indirect association between a positive sense of mathematics and reflective self-awareness (Lee, 2005; Schaaf et al., 2013).

The multiple mediation findings from this research offer critical pedagogical implications for the integration of cognitive and affective processes in mathematics education (Hannula, 2002). The finding that the dialectical dimension of critical thinking tendency emerges as a prominent mediator in the model suggests that a key mechanism linking students’ affective mathematical inputs to higher-level problem-solving and reflective thinking skills may be their ability to think “flexibly and multidimensionally” (Sternberg, 2003). Conversely, the failure of the analytical dimension (breaking down data, applying formulas and rules), which is often central to traditional mathematics teaching, to assume a significant mediating role in the model indicates that analytical effort alone is insufficient to bridge affective characteristics to reflective skills (Schoenfeld, 2016). Therefore, to stimulate reflective thinking (inquiry, reasoning, and evaluation) in mathematics classrooms (Phan, 2010), instead of focusing solely on dogmatic and prescriptive analytical steps, it is considered essential to design dialectical learning environments where students can experience cognitive contradictions, synthesize alternative solutions, and operate thesis-antithesis processes.

The research findings reveal that the direct effect of affective entry characteristics toward mathematics on reflective thinking skills in problem-solving (inquiry, reasoning, and evaluation) is partially and sequentially mediated through the sub-dimensions of critical thinking disposition. When the hierarchical structure of the model is examined, it is observed that the most potent indirect effect of AECM on all three sub-dimensions of reflective thinking, which also possesses a high effect size, occurs solely through Dialectical Thinking (Y1). This indicates that students with positive affective characteristics in mathematical processes primarily activate their tendencies to synthesize and question different perspectives, which in turn directly nurtures their inquiry, reasoning, and evaluation skills in problem-solving. On the other hand, although the sequential multiple mediation pathway (AECM → Y1 → Y2 → Z1, Z2, Z3)—where Dialectical Thinking and Disposition (Y2) variables are sequentially activated—is statistically significant, it is noteworthy that the effect size drastically diminishes within this chain-like process. More importantly, the fact that no indirect pathway involving the Analysis (Y3) sub-dimension, which represents the analytical thinking structure, yielded significant results—and even exhibited negative trends in certain relationships—should not be interpreted as a definitive pedagogical conclusion. Indeed, the high inter-correlations among the sub-dimensions of critical and reflective thinking suggest that a collinearity or statistical suppression effect may have occurred within the model. Furthermore, potential losses of nuance during the linguistic or conceptual translation processes of the measurement instrument’s items, or the specific psychometric characteristics of the scale’s factor structure within this particular sample, might have obscured the actual function of the Analysis (Y3) dimension. Therefore, the lack of support for the mediating role of the analytical sub-dimension should be evaluated as a reflection of methodological and statistical limitations rather than a pedagogical deficiency, and it should be re-tested in future studies using alternative measurement instruments.

## 5. Limitations and Future Suggestions

One of the main limitations of this research is that the data collection process was conducted entirely using Likert-type scales based on participants’ self-reports. While this reflects students’ perceptions of reflective thinking, critical thinking tendency, and affective input characteristics, it carries the risk of not fully matching students’ actual behavioral patterns. Future studies are recommended to use mixed-methods approaches, incorporating qualitative data collection tools such as clinical interviews, think-aloud protocols during problem-solving, or direct observation, in addition to quantitative data, to examine these latent structures more deeply.

Secondly, significant sample limitations restrict the generalizability of our structural model. The study sample consisted only of middle school students attending public schools in a specific geographic region. Socioeconomic variables, private school dynamics, and structural differences in regional educational subcultures were not controlled for or compared. Furthermore, the data collection process was limited to specific grade levels, ignoring developmental changes that may occur across the broad K-12 spectrum. To enhance the external validity of the structural model, future studies should utilize cross-sectional or longitudinal designs that also include private educational institutions and trace how the mediating pathway evolves as students become socio-cognitively mature.

The fact that the study’s data collection period coincided with the post-COVID-19 pandemic may have affected the temporal nature of the findings. Compared to studies conducted in the pre-pandemic period, it can be said that the affective adjustment processes and critical thinking mechanisms of students transitioning from distance learning to in-person learning were affected by this crisis period. This can be considered a structural limitation of the study; since the lack of longitudinal data from the pre-pandemic period makes it difficult to clearly isolate the full impact of the pandemic. In future studies, it is recommended that meta-analyses or comparative studies directly comparing data from the pre-pandemic period with post-pandemic data be conducted to understand the long-term impact of educational crises on these cyclical relationships.

Considering the final outcome of this study, mathematics teachers should realize that directly forcing a student with weak math attitudes into a deep reflective error analysis is not very effective pedagogically. Teachers should actively target critical thinking tendency, which is a mediating variable, in their teaching strategies. Lessons should begin by reconstructing the student’s affective self-concept through structured tasks, followed immediately by critical thinking tendency interventions. This process can directly activate the cognitive bridge needed to unlock deep and self-improving reflective thinking.

Furthermore, in parallel with the relationship between higher-order cognitive skills and academic and professional success (Bakır & Eğmir, 2022; Scott & Markert, 1994), mathematics curricula should treat emotion, critical thinking tendency, and reflection as an inseparable, sequential line. Assessment frameworks should evaluate not only the final product but also the student’s journey along this line; they should utilize critical-reflective portfolios that track how their emotional confidence translates into analytical questioning and retrospective mastery.

## Figures and Tables

**Table 1:** Distribution of the Participants by Demographic Characteristics (*N* = 649).

Variables		*N*	*%*
**Gender**	Girl	358	55.2
	Boy	291	44.8
**Grade level**	5	168	25.9
	6	159	24.5
	7	166	25.6
	8	156	24.0

**Table 2:** Descriptive statistics and Pearson’s correlations between the study variables.

Variables	M	SD	1	2	3	Kurtosis	Skewness
(1) Affective Entry Characteristics Toward Mathematics	54.448	14.659	-	0.498 **	0.582 **	−0.663	−0.123
(2) Critical Thinking Tendency	80.120	13.685	0.498 **	-	0.726 **	0.850	−0.614
(3) Reflective Thinking Skills For Problem Solving	51.736	10.972	0.582 **	0.726 **	-	0.155	−0.536

** *p* < .01.

**Table 3:** Bootstrapping results for the mediation model.

Pattern Paths	Bootstrap Values Coefficient	SE	t	95% CI Lower	Upper	K^2^
Model without mediator						
Total effect AECM → RTSPS	0.4353	0.0239	18.182	0.3883	0.4823	0.2894
Model with critical thinking tendency as the mediator						
Direct effect AECM → RTSPS	0.2187	0.0217	10.076	0.1761	0.2613	
Indirect effect of CTT AECM → CTT → RTSPS	0.2166	0.0178		0.1815	0.2525	

SE: Standard Error; K^2^ = Fully standardized size of indirect effect (mediation effect); AECM: Affective Entry Characteristics toward Mathematics; CTT: Critical Thinking Tendency; RTSPS: Reflective Thinking Skills for Problem Solving.

**Table 4:** Total, Direct, and All Specific Indirect Effect Coefficients of the Models.

RTSPS	Path/Type of Effect	Bootstrap Values Coefficient	SE	t	BootLLCI	BootULCI	K^2^
Z1	Total effect (AECM → Z1)	0.1497	0.0097	15.42 ***	0.1306	0.1687	0.3004
	Direct effect (AECM → Z1)	0.0630	0.0095	6.64 ***	0.0443	0.0816	
	Indirect effect	0.0867	0.0075	—	0.0721	0.1017	
	AECM → Y1 → Z1	0.0716	0.0078	—	0.0567	0.0871	0.2479
	AECM → Y2 → Z1	0.0032	0.0022	—	−0.0006	0.0080	
	AECM → Y3 → Z1	−0.0003	0.0006	—	−0.0019	0.0007	
	AECM → Y1 → Y2 → Z1	0.0093	0.0026	—	0.0044	0.0145	0.0321
	AECM → Y1 → Y3 → Z1	0.0026	0.0036	—	−0.0044	0.0099	
	AECM → Y2 → Y3 → Z1	0.0001	0.0002	—	−0.0003	0.0007	
	AECM → Y1 → Y2 → Y3 → Z1	0.0004	0.0005	—	−0.0007	0.0015	
Z2	Total effect (AECM → Z2)	0.1566	0.0096	16.28 ***	0.1377	0.1755	0.3010
	Direct effect (AECM → Z2)	0.0691	0.0093	7.43 ***	0.0509	0.0874	
	Indirect effect	0.0874	0.0071	—	0.0741	0.1018	
	AECM → Y1 → Z2	0.0721	0.0077	—	0.0573	0.0880	0.2483
	AECM → Y2 → Z2	0.0022	0.0016	—	−0.0004	0.0060	
	AECM → Y3 → Z2	−0.0008	0.0008	—	−0.0028	0.0004	
	AECM → Y1 → Y2 → Z2	0.0065	0.0024	—	0.0020	0.0112	0.0222
	AECM → Y1 → Y3 → Z2	0.0062	0.0036	—	−0.0008	0.0136	
	AECM → Y2 → Y3 → Z2	0.0003	0.0003	—	−0.0001	0.0011	
	AECM → Y1 → Y2 → Y3 → Z2	0.0009	0.0006	—	−0.0001	0.0021	
Z3	Total effect (AECM → Z3)	0.1290	0.0078	16.57 ***	0.1137	0.1443	0.2786
	Direct effect (AECM → Z3)	0.0632	0.0078	8.09 ***	0.0478	0.0785	
	Indirect effect	0.0659	0.0060	—	0.0546	0.0782	
	AECM → Y1 → Z3	0.0558	0.0063	—	0.0438	0.0687	0.2359
	AECM → Y2 → Z3	0.0018	0.0014	—	−0.0003	0.0052	
	AECM → Y3 → Z3	−0.0004	0.0006	—	−0.0019	0.0005	
	AECM → Y1 → Y2 → Z3	0.0053	0.0021	—	0.0013	0.0095	0.0224
	AECM → Y1 → Y3 → Z3	0.0028	0.0031	—	−0.0032	0.0091	
	AECM → Y2 → Y3 → Z3	0.0001	0.0002	—	−0.0002	0.0007	
	AECM → Y1 → Y2 → Y3 → Z32	0.0004	0.0005	—	−0.0005	0.0014	

*** *p* < .001.

## Data Availability

The datasets analyzed or generated during the current study are available from the corresponding author upon reasonable request.

## Appendix A

**Table A1:** Ortaokul Öğrencileri İçin Eleştirel Düşünme Eğilimi Ölçeği [Critical Thinking Tendency Scale].

	HER ZAMAN	SIK SIK	BAZEN	NADİREN	HİÇ
1. Çevremdeki insanların fikirlerini dikkatlice dinlerim.	5	4	3	2	1
2. İnsanların fikirlerini oluşturan nedenleri önemserim.	5	4	3	2	1
3. Problemlere ürettiğim çözümleri paylaşırken kendime güvenirim.	5	4	3	2	1
4. Karşıt fikirleri değerlendirirken tarafsız davranırım.	5	4	3	2	1
5. Bir durumu tüm yönleri ile tanımlayabilirim.	5	4	3	2	1
6. Fikirlerimin arkasında durabilirim.	5	4	3	2	1
7. Bir probleme getirdiğim çözümün neden doğru olduğunu açıklayabilirim.	5	4	3	2	1
8. Bilgiyi edinmeye meraklıyımdır.	5	4	3	2	1
9. Kendi düşüncelerimin farkındayımdır.	5	4	3	2	1
10. İnsanlarla herhangi bir konu üzerinde tartışmalarda bulunmayı severim.	5	4	3	2	1
11. Bir cümledeki ön yargıyı sezebilirim.	5	4	3	2	1
12. Bir olayın temelindeki nedenleri keşfetmek benim için önemlidir.	5	4	3	2	1
13. Çevremdeki insanların fikirlerini tarafsız bir şekilde dinlerim.	5	4	3	2	1
14. Problem çözme konusunda kendime güvenirim.	5	4	3	2	1
15. Mantık hatalarını tanımlayabilirim.	5	4	3	2	1
16. Düşünce sürecimde temel amacım doğrulara ulaşmaktır.	5	4	3	2	1
17. Olayların detaylarına odaklanabilirim.	5	4	3	2	1
18. Eleştirel düşünmeyi severim.	5	4	3	2	1
19. Günlük hayatta eleştirel düşünmeye başvururum.	5	4	3	2	1
20. Eleştirel düşünme benim için önemlidir.	5	4	3	2	1
21. Eleştirel düşünmenin bana katkı sağladığını düşünüyorum.	5	4	3	2	1

**Table A2:** Matematiğe Yönelik Duyuşsal Giriş Özellikleri [Affective Entry Characteristics Toward Mathematics Scale].

	Kesinlikle Katılıyorum	Katılıyorum	Katılmıyorum	Kesinlikle Katilmiyorum
1. Matematik çalışmak benim için kolaydır.				
2. Diğer derslerle karşılaştırdığımda matematik dersindeki yeteneğim çok yüksektir.				
3. Matematik dersinde diğer derslerden daha mutlu olurum.				
4. Matematikte iyiyimdir.				
5. Matematik dersinde konuları çabucak öğrenirim.				
6. Matematik dersini gerçekten severim.				
7. Matematik insanların öğrenmesi gereken en önemli derslerden birisidir.				
8. Matematik problemlerini çözmek benim için kolaydır.				
9. Matematik dersinin zorluğu hoşuma gider.				
10. Bana göre, matematik dersindeki başarım, sınıf ortalamasının çok üstünde olacak.				
11. Matematik dersine isteyerek gelirim.				
12. Matematik yeteneği açısından sınıfta en iyiler arasındayım.				
13. Matematik dersinde en zor konuyu bile anlarım.				
14. Matematikte her zaman başarılı olmuşumdur.				
15. Matematik dersini sabırsızlıkla beklerim.				
16. Matematiğe ilgi duyarım.				
17. Matematik dersinden keyif alırım.				
18. Matematik dersindeki konuları öğrenebileceğim konusunda kendime güvenirim.				
19. Matematiğin her zaman en iyi derslerimden biri olduğunu düşünmüşümdür.				
20. Matematik dersine çalışmak için zaman ayırırım.				

**Table A3:** Problem Çözmeye Yönelik Yansıtıcı Düşünme Becerisi Ölçeği [Reflective Thinking Skills For Problem Solving Scale].

	Her Zaman	Çoğu Zaman	Bazen	Nadiren	Hiçbir Zaman
(1) Bir problemi çözemediğimde, neden çözemediğimi anlamak için kendime sorular sorarım.					
(2) Problemi çözdükten sonra daha iyi bir çözüm yolu bulabilir miyim diye düşünürüm.					
(3) Arkadaşlarımın çözüm yollarını sorgulayarak daha iyi bir yol bulmaya çalışırım.					
(4) Çözüm yollarımı tekrar tekrar değerlendirip bir sonraki problemi daha iyi çözmeye çalışırım.					
(5) Problem çözerken, hangi işlemi neden yaptığımı düşünerek yaparım.					
(6) Bir problemi çözdüğümde, yaptığım işlemleri tekrar inceler, değerlendiririm.					
(7) Problem çözerken, farklı çözüm yolları bulmak için kendime sorular sorarım.					
(8) Problem çözerken, yaptığım işlemlerin nedenini düşünerek, bulduğum sonuçla ilişkisini kurmaya çalışırım.					
(9) Bir problemi okuduğumda, çözüm için hangi bilgiye ihtiyacım olduğunu düşünürüm.					
(10) Problemi çözüp sonucunu bulduktan sonra yaptığım işlemleri kontrol ederim.					
(11) Bir problemi okuduğumda, daha önce çözdüğüm problemleri düşünerek benzerlik ve farklılıklarına göre aralarında ilişki kurarım.					
(12) Problem çözerken, her işlemimi önceki ve sonraki adımlarımı düşünerek yaparım.					
(13) Problemi okuduğumda verilen ve istenenleri belirlemek için kendime sorular sorarım.					
(14) Problemi çözdükten sonra arkadaşlarımın çözümleri ile karşılaştırır, sonucumu değerlendiririm.					

## References

[B1-jintelligence-14-00149] Afshar H. S., Rahimi M. (2016). Reflective thinking, emotional intelligence, and speaking ability of EFL learners: Is there a relation?. Thinking Skills and Creativity.

[B2-jintelligence-14-00149] Anderson L. W., Bourke S. F. (2000). Assessing affective characteristics in schools.

[B3-jintelligence-14-00149] Arpin H., Mirza A., Astuti D. (2015). Pengaruh tingkat kecemasan matematika terhadap kemampuan berpikir kritis siswa kelas x sma. Sainsmat.

[B4-jintelligence-14-00149] Aryani F., Rais M., Wirawan H. (2017). Reflective learning model in improving student critical thinking skills. Global Journal of Engineering Education.

[B5-jintelligence-14-00149] Ashcraft M. H., Kirk E. P. (2001). The relationships among working memory, math anxiety, and performance. Journal of Experimental Psychology: General.

[B6-jintelligence-14-00149] Bakır T., Eğmir E. (2022). Examination of the relationship between secondary school students’ critical thinking dispositions and metacognitive awareness. e-International Journal of Educational Research.

[B7-jintelligence-14-00149] Baron R. M., Kenny D. A. (1986). The moderator-mediator variable distinction in social psychological research: Conceptual, strategic, and statistical considerations. Journal of Personality and Social Psychology.

[B8-jintelligence-14-00149] Bloom B. S., Özçelik D. A. (1998). İnsan nitelikleri ve okulda öğrenme *[Human qualities and learning at school]*.

[B9-jintelligence-14-00149] Bruning R., Schraw G. J., Ronning R. R. (1995). Cognitive psyhology and instruction.

[B10-jintelligence-14-00149] Chamdani M., Yusuf F. A., Salimi M., Fajari L. E. W. (2022). Meta-analysis study: The relationship between reflective thinking and learning achievement. Journal on Efficiency and Responsibility in Education.

[B11-jintelligence-14-00149] Creswell J. W. (2012). Educational research: Planning, conducting, evaluating, quantitative and qualitative research.

[B12-jintelligence-14-00149] Çalışkan M., Serçe H. (2016). Matematiğe yönelik duyuşsal giriş özellikleri ölçeği: Geçerlik ve güvenirlik çalışması [Affective entry characteristics scale for mathematics: A study of reliability and validity]. International Journal of Eurasia Social Sciences.

[B13-jintelligence-14-00149] Daud D., Santoso R. H. (2018). Device learning development using cabri 3D with problem-solving method based on oriented critical thinking ability and learning achievements of junior high school students. 5th Asia pacific education conference (AECON 2018). Advances in Social Science, Education and Humanities Research (ASSEHR).

[B14-jintelligence-14-00149] Dubinsky E. (2002). Reflective abstraction in advanced mathematical thinking. Advanced mathematical thinking.

[B15-jintelligence-14-00149] Edirmanasinghe N. (2020). Using youth participatory action research to promote self-efficacy in and science. Professional School Counseling.

[B16-jintelligence-14-00149] Ennis R. H. (1985). A logical basis for measuring critical thinking skills. Educational Leadership.

[B17-jintelligence-14-00149] Ennis R. H. (1991). Critical thinking: A streamlined conception. Teaching Philosophy.

[B18-jintelligence-14-00149] Erdogan F. (2019). Effect of cooperative learning supported by reflective thinking activities on students’ critical thinking skills. Eurasian Journal of Educational Research.

[B19-jintelligence-14-00149] Evans J. D. (1996). Straightforward statistics for the behavioral sciences.

[B20-jintelligence-14-00149] Facione P. A., Facione N. C. (1992). The california critical thinking skills test. Forms a and b–test manual.

[B21-jintelligence-14-00149] Farnila Y., Johar R., Usman U. (2021). Students’ reflective thinking process in mathematical problem-solving reviewed from self-confidence. AIP Conference Proceedings.

[B22-jintelligence-14-00149] Field A. P. (2005). Is the Meta-Analysis of Correlation Coefficients Accurate When Population Correlations Vary?. Psychological Methods.

[B23-jintelligence-14-00149] Firdaus, Kailani I., Bakar M. N. B., Bakry B. (2015). Developing critical thinking skills of students in mathematics learning. Journal of Education and Learning (EduLearn).

[B24-jintelligence-14-00149] Fisher A., Scriven M. (1997). Critical thinking its definition and assessment.

[B25-jintelligence-14-00149] Ganley C. M., Lubienski S. T. (2016). Mathematics confidence, interest, and performance: Examining gender patterns reciprocal relations. Learning and Individual Differences.

[B26-jintelligence-14-00149] George D., Mallery M. (2010). SPSS for windows step by step: A simple guide and reference, 17.0 update.

[B27-jintelligence-14-00149] Ghanizadeh A. (2017). The interplay between reflective thinking, critical thinking, self-monitoring, and academic achievement in higher education. Higher Education: The International Journal of Higher Education Research.

[B28-jintelligence-14-00149] Göğüş A., Göğüş N. G., Bahadır E. (2020). Intersections between critical thinking skills and reflective thinking skills toward problem solving. Pamukkale Üniversitesi Eğitim Fakültesi Dergisi.

[B29-jintelligence-14-00149] Halpern D. F. (1998). Teaching critical thinking for transfer across domains dispositions, skills structure training, and metacognitive monitoring. American Psychologist.

[B30-jintelligence-14-00149] Halpern D. F. (2014). Thought and knowledge: An introduction to critical thinking.

[B31-jintelligence-14-00149] Halpern D. F., Dunn D. S. (2021). Critical thinking: A model of intelligence for solving real-world problems. Journal of Intelligence.

[B32-jintelligence-14-00149] Hannula M. S. (2002). Attitude towards mathematics: Emotions, expectations and values. Educational Studies in Mathematics.

[B33-jintelligence-14-00149] Hayes A. F. (2009). Beyond baron and kenny: Statistical mediation analysis in the new millennium. Communication Monographs.

[B34-jintelligence-14-00149] Hayes A. F. (2017). Introduction to mediation, moderation, and conditional process analysis: A regression-based approach.

[B35-jintelligence-14-00149] Hayes A. F. (2022). Introduction to mediation, moderation, and conditional process analysis: A regression-based approach.

[B36-jintelligence-14-00149] Hong Y. C., Choi I. (2015). Assessing reflective thinking in solving design problems: The development of a questionnaire. British Journal of Educational Technology.

[B37-jintelligence-14-00149] Inoue N., Buczynski S. (2011). You asked open-ended questions, now what? understanding the nature of stumbling blocks in teaching inquiry lessons. The Mathematics Educator.

[B38-jintelligence-14-00149] Kazemi S., Ashraf H., Motallebzadeh K., Zeraatpishe M., Piro J. S. (2020). Development and validation of a null curriculum questionnaire focusing on 21st century skills using the Rasch model. Cogent Education.

[B39-jintelligence-14-00149] Kember D. (2010). Determining the level of reflective thinking from students’ written journals using a coding scheme based on the work of mezirow. International Journal of Lifelong Education.

[B40-jintelligence-14-00149] Kholid M., Sa’dijah C., Hidayanto E., Permadi H. (2020). How are students’ reflective thinking for problem solving?. Journal for the Education of Gifted Young Scientists.

[B41-jintelligence-14-00149] King P. M., Kitchener K. S. (1994). Developing reflective judgment: Understanding and promoting intellectual growth and critical thinking in adolescents and adults.

[B42-jintelligence-14-00149] Kızılkaya G., Aşkar P. (2009). Problem çözmeye yönelik yansıtıcı düşünme becerisi ölçeğinin geliştirilmesi [The development of a reflective thinking skill scale towards problem solving]. Education and Science.

[B43-jintelligence-14-00149] Kramaski B., Weiss I., Sharon S. (2013). Generic versus context-specific prompts for supporting self-regulation in mathematical problem solving among students with low or high prior knowledge. Journal of Cognitive Education and Psychology.

[B44-jintelligence-14-00149] Kuncoro K. S., Hidayat A. (2025). Thinking beyond the equations: A deep dive into reflective thinking for mathematics learning. Participatory Educational Research (PER).

[B45-jintelligence-14-00149] Lee H. J. (2005). Understanding and assessing preservice teachers’ reflective thinking. Teaching and Teacher Education.

[B46-jintelligence-14-00149] León J. M. (2015). A baseline study of strategies to promote critical thinking in the preschool classroom (un estudio de base sobre estrategias para la promoción de pensamiento critico en las aulas de preescolar). GIST Education and Learning Research Journal.

[B47-jintelligence-14-00149] Leung D. Y. P., Kember D. (2003). The relationship be-tween approaches to learning and reflection upon practice. Educational Psychology.

[B48-jintelligence-14-00149] Lewis A., Smith D. (1993). Defining higher order thinking. Theory into Practice.

[B49-jintelligence-14-00149] Liu Y., Páztor A. (2022). Effects of problem-based learning instructional intervention on critical thinking in higher education: A meta-analysis. Thinking Skills and Creativity.

[B50-jintelligence-14-00149] Maass J., Schlöglmann W. (2009). Beliefs and attitudes in mathematics education: New research results.

[B51-jintelligence-14-00149] MacKinnon D. P., Lockwood C. M., Williams J. (2004). Confidence limits for the indirect effect: Distribution of the product and resampling methods. Multivariate Behavioral Research.

[B52-jintelligence-14-00149] Madadkhani Z., Nikoogoftar M. (2015). Critical thinking in nurses: Predictive role of emotional intelligence. Journal of Hayat.

[B53-jintelligence-14-00149] Maloney E. A., Retanal F. (2020). Higher math anxious people have a lower need for cognition and are less reflective in their thinking. Acta Psychologica.

[B54-jintelligence-14-00149] Manalo E., Kusumi T., Koyasu M., Michita Y., Tanaka Y. (2013). To what extent do culture-related factors influence university students’ critical thinking use?. Thinking Skills and Creativity.

[B55-jintelligence-14-00149] Merkebu J., Kitsantas A., Durning S. J., Ma T. (2023). What is metacognitive reflection? The moderating role of metacognition on emotional regulation and reflection. Frontiers in Education.

[B56-jintelligence-14-00149] Merriam S. B. (2009). Qualitative research: A guide to design and implementation.

[B57-jintelligence-14-00149] Mezirow J. (1991). Transformative dimensions of adult learning.

[B58-jintelligence-14-00149] Najafi M., Motlagh M. K., Najafi M., Kashani A. S., Zarghi N., Shirazi M. (2022). Trend of tendency to critical thinking among medical students in tehran university of medical sciences, 2010–2015: A longitudinal study. Journal of Education and Health Promotion.

[B59-jintelligence-14-00149] National Council of Teachers of Mathematics [NCTM] (2020). Catalyzing change in middle school mathematics: Initiating critical conversations.

[B60-jintelligence-14-00149] Nosich M. G., Aybek B. (2012). Eleştirel düşünme ve disiplinlerarası eleştirel düşünme rehberi *[A guide to critical thinking and interdisciplinary critical thinking]*.

[B61-jintelligence-14-00149] Ormrod J. E., Baloğlu M. (2018). Öğrenme psikolojisi *[Psychology of learning]*.

[B62-jintelligence-14-00149] Özçelik D. A. (1998). Eğitim programları ve öğretim *[Educational programs and teaching]*.

[B63-jintelligence-14-00149] Pantziara M., Philippou G. N. (2015). Students’ motivation in the mathematics classroom. revealing causes and consequences. International Journal of Science and Mathematics Education.

[B64-jintelligence-14-00149] Papanastasiou C. (2002). Effects of background and school factors on the mathematics achievement. Educational research and evaluation. An International Journal on Theory and Practice.

[B65-jintelligence-14-00149] Paul R. (1990). Critical thinking.

[B66-jintelligence-14-00149] Paul R., Elder L., Aslan E., Sart G. (2013). Kritik düşünce *[Critical thinking]*.

[B67-jintelligence-14-00149] Pavitola L., Rieksta R. (2025). Critical thinking skills in mathematical proof tasks in the context of quality education: Case study. Eurasia Journal of Mathematics, Science and Technology Education.

[B68-jintelligence-14-00149] Phan H. P. (2009). Exploring students’ reflective thinking practice, deep processing strategies, effort, and achievement goal orientations. Educational Psychology.

[B69-jintelligence-14-00149] Phan H. P. (2010). Critical thinking as a self-regulatory process component in teaching and learning. Psicothema.

[B70-jintelligence-14-00149] Preacher K. J., Hayes A. F. (2004). SPSS and SAS procedures for estimating indirect effects in simple mediation models. Behavior Research Methods, Instruments and Computers.

[B71-jintelligence-14-00149] Preacher K. J., Kelley K. (2011). Effect size measures for mediation models: Quantitative strategies for communicating indirect effects. Psychological Methods.

[B72-jintelligence-14-00149] Preacher K. J., Rucker D. D., Hayes A. F. (2007). Addressing moderated mediation hypotheses: Theory, methods, and prescriptions. Multivariate Behavioral Research.

[B73-jintelligence-14-00149] Procter L. (2020). Fostering critically reflective thinking with first-year university students: Early thoughts on implementing a reflective assessment task. Reflective Practice.

[B74-jintelligence-14-00149] Ramdani R., Syamsuddin A., Sirajuddin S. (2019). Development of mathematical module problem solving approach to train student’s reflective thinking. Pedagogical Research.

[B75-jintelligence-14-00149] Rodd J. (1999). Encouraging young children’s critical and creative thinking skills: An approach in one English elementary school. Childhood Education.

[B76-jintelligence-14-00149] Rodgers C. (2002). Defining reflection: Another look at john dewey and reflective thinking. Teachers College Record.

[B77-jintelligence-14-00149] Samuelsson J. (2023). Developing students’ relationships with mathematics. Educational Action Research.

[B78-jintelligence-14-00149] Sasson I., Yehuda I., Malkinson N. (2018). Fostering the skills of critical thinking and question-posing in a project-based learning environment. Thinking Skills and Creativity.

[B79-jintelligence-14-00149] Sayaf A. M., Alamri M. M., Alqahtani M. A., Alrahmi W. M. (2022). Factors influencing university students’ adoption of digital learning technology in teaching and learning. Sustainability.

[B80-jintelligence-14-00149] Schaaf M. V., Baartman L., Prins F., Oosterbaan A., Schaap H. (2013). Feedbackdialogues that stimulate students’ reflective thinking. Scandinavian Journal of Educational Research.

[B81-jintelligence-14-00149] Scheibe D. A., Was C. A., Dunlosky J., Thompson C. A. (2023). Metacognitive cues, working memory, and math anxiety: The regulated attention in mathematical problem solving (ramps) framework. Journal of Intelligence.

[B82-jintelligence-14-00149] Schoenfeld A. H. (2016). Mathematical problem solving.

[B83-jintelligence-14-00149] Scott J. N., Markert R. J. (1994). Relationship between critical thinking skills and success in preclinical courses. Academic Medicine.

[B84-jintelligence-14-00149] Seel N. M., Seel N. M. (2012). Bloom’s model of school learning. Encyclopedia of the sciences of learning.

[B85-jintelligence-14-00149] Sevgi S., Alpaslan A. (2020). Ortaokul öğrencilerinde matematiğe yönelik duyuşsal giriş özellikleri ile sayı duyusuna yönelik öz yeterlikleri incelenmesi [Investigation of middle school students’ affective entry characteristics for mathematics and number sense self-efficacy]. Turkish Journal of Educational Studies.

[B86-jintelligence-14-00149] Shermis S. S. (1992). Critical thinking: Helping students learn reflectively.

[B87-jintelligence-14-00149] Sternberg R. J. (1999). Nature of cognition.

[B88-jintelligence-14-00149] Sternberg R. J. (2003). Wisdom, intelligence, and creativity synthesized.

[B89-jintelligence-14-00149] Su H. F., Ricci F. A., Mnatsakanian M. (2016). Mathematical teaching strategies: Pathways to critical thinking and metacognition. Journal of Research in Education and Science (IJRES).

[B90-jintelligence-14-00149] Suárez J. R., Pabón D., Villavez L., Martin J. A. (2018). Critical thinking and philosophy.

[B91-jintelligence-14-00149] Tall D., Kuncoro K. S., Hidayat A., Nadzeri M. B., Hendriyanto A., Meirani M., Juandi D. (2002). The psychology of advanced mathematical thinking. Advanced participatory educational research (PER)-333-thinking beyond the equations: A deep mathematical thinking.

[B92-jintelligence-14-00149] Ünver G. (2003). Yansıtıcı düşünme *[Reflective thinking]*.

[B93-jintelligence-14-00149] Vendrell M., Rodríguez J. M. (2020). Critical thinking: Conceptualization and relevance within higher education. Journal of Higher Education.

[B94-jintelligence-14-00149] Wolters C. A. (2003). Regulation of motivation: Evaluating an underemphasized aspect of self-regulated learning. Educational Psychologist.

[B95-jintelligence-14-00149] Yıldırım Döner S., Demir S. (2022). Ortaokul öğrencileri için eleştirel düşünme eğilimi ölçeği’nin geliştirilmesi: Geçerlik ve güvenirlik çalışması [Developing the critical thinking disposition scale for secondary school students: A validity and reliability study]. Pamukkale University Journal of Education.

[B96-jintelligence-14-00149] Zhang L. F. (2003). Contributions of thinking styles to critical thinking dispositions. The Journal of Psychology.

[B97-jintelligence-14-00149] Zhao X., Lynch J. G., Chen Q. (2010). Reconsidering baron and kenny: Myths and truths about mediation analysis. Journal of Consumer Research.

[B98-jintelligence-14-00149] Zimmerman B. J. (2000). Self-efficacy: An essential motive to learn. Contemporary Educational Psychology.

